# Gemcitabine elaidate and ONC201 combination therapy for inhibiting pancreatic cancer in a KRAS mutated syngeneic mouse model

**DOI:** 10.1038/s41420-024-01920-9

**Published:** 2024-03-29

**Authors:** Virender Kumar, Bharti Sethi, Dalton W. Staller, Prakash Shrestha, Ram I. Mahato

**Affiliations:** 1 Department of Pharmaceutical Sciences, University of Nebraska Medical Center, Omaha, NE 68198 USA; 2 Department of Cellular & Integrative Physiology, University of Nebraska Medical Center, Omaha, NE USA

**Keywords:** Preclinical research, Endocrine cancer

## Abstract

Approximately 90% of pancreatic cancer (PC) contain KRAS mutations. Mutated KRAS activates the downstream oncogenic PI3K/AKT and MEK signaling pathways and induces drug resistance. However, targeting both pathways with different drugs can also lead to excessive toxicity. ONC201 is a dual PI3K/AKT and MEK pathway inhibitor with an excellent safety profile that targets death receptor 5 (DR5) to induce apoptosis. Gemcitabine (GEM) is a first-line chemotherapy in PC, but it is metabolically unstable and can be stabilized by a prodrug approach. In this study, phospho-Akt, phospho-mTOR, and phospho-ERK protein expressions were evaluated in patient PDAC-tissues (*n* = 10). We used lipid-gemcitabine (L_GEM) conjugate, which is more stable and enters the cells by passive diffusion. Further, we evaluated the efficacy of L_GEM and ONC201 in PC cells and “KrasLSL-G12D; p53LoxP; Pdx1-CreER (KPC) triple mutant xenograft tumor-bearing mice. PDAC patient tissues showed significantly higher levels of p-AKT (Ser473), p-ERK (T202/T204), and p-mTOR compared to surrounding non-cancerous tissues. ONC201 in combination with L_GEM, showed a superior inhibitory effect on the growth of MIA PaCa-2 cells. In our in-vivo study, we found that ONC201 and L_GEM combination prevented neoplastic proliferation via AKT/ERK blockade to overcome chemoresistance and increased T-cell tumor surveillance. Simultaneous inhibition of the PI3K/AKT and MEK pathways with ONC201 is an attractive approach to potentiate the effect of GEM. Our findings provide insight into rational-directed precision chemo and immunotherapy therapy in PDAC.

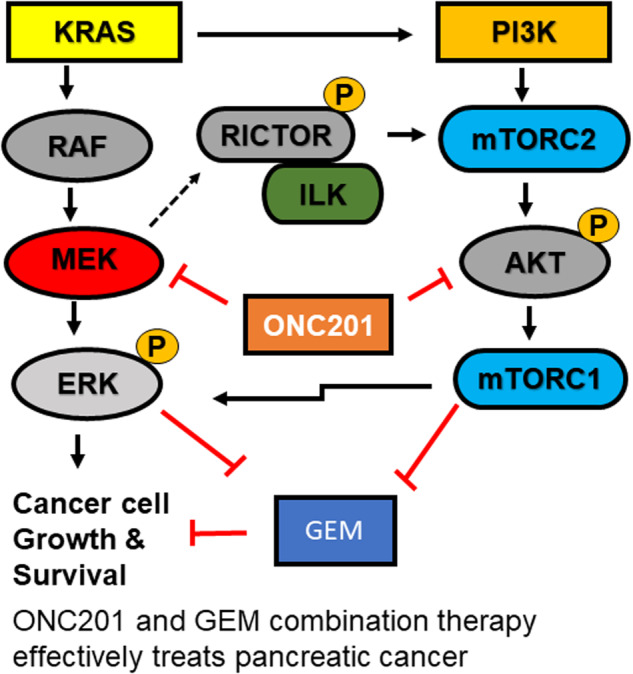

## Introduction

Pancreatic ductal adenocarcinoma (PDAC) is one of the most difficult-to-treat cancers. The anti-metabolite drug gemcitabine (GEM) is the gold standard and the first-line of treatment for PDAC [1]. GEM is a prodrug that is converted stepwise to two active metabolites by deoxycytidine kinase, GEM diphosphate (dFdCDP), and GEM triphosphate (dFdCTP) [2]. The dFdCDP depletes the cells’ deoxyribonucleotide (dNTP) pools via inhibition of ribonucleotide reductase 1 (RRM1) [3] Whereas the dFdCTP competes with endogenous dNTPs for incorporation into DNA and kill the cells. However, the GEM response rate is low, and overall survival has only improved minimally in PDAC [4]. The main reason for this failure is its intrinsic instability due to rapid metabolism in the liver and acquired resistance [5]. Further, the intracellular uptake of GEM is affected by variable expression of human equilibrate nucleoside transporter-1 (hENT-1) receptors in PC [6]. Therefore, several efforts have been made to improve the overall performance of GEM in recent years. Various modifications have already been performed on the 4-(N)- and 5′-positions of GEM, such as the incorporation of poly(ethylene glycol) (PEG), valproic acid, 1,1′, 2-tris-nor-squalene acid (squalene) or valeroyl, heptanoyl, lauroyl, stearoyl linear acyl derivates in the 4-(*N*) site, and the addition of fatty acid chains or phosphate-function-protecting groups to the 5′-position [7] to improve its chemical stability. Gemcitabine-5′-elaidate (CP-4126, CO-101) is a one of such GEM prodrugs consisting of a fatty acid derivative (abbreviated as L_GEM). L_GEM exerts high anticancer activity and enters tumor cells independent of hENT1 making it superior to other GEM analogs [8]. Further, it was shown that L_GEM is not a substrate of GEM metabolizing enzyme cytidine deaminase (CDA) and retains its activity for longer in vivo [9]. Despite all these qualities, clinical study did not show an increased efficacy of L_GEM in PC patients, indicating intrinsic resistance. Simultaneous activation of the PI3K and ERK pathways has been suggested to generate intrinsic resistance to GEM in PC patients [10–12]. Another strategy to enhance GEM efficacy is combination therapy with other drugs. In previous studies from our group, GEM was combined with vismodegib, and miR-519c or miR-205, to assess their joined effect on cancerous cells [6, 13].

ONC201 is a new member of the imipridone class of anticancer small molecules that primarily target dopamine receptor 2 (DR2) and TNF-related apoptosis-inducing ligand (TRAIL)—inducing activity [14]. ONC201 also inhibits the DR5 pathway, which is upregulated in PC and correlates with poor prognosis in patients. DR5 targeting monoclonal antibody AMG 655 (conatumumab) has previously been investigated in a phase 2 clinical trial for treating PC in combination with GEM [15]. Further, it has been suggested that cancer stem cells (CSCs) are responsible for tumor relapse and are highly resistant to chemotherapy and radiation [16]. ONC201 has also demonstrated efficacy in eliminating CSCs in colorectal cancer, glioblastoma, and prostate cancer models [17–19]. ONC201 also inactivates AKT/ERK signaling in tumor cells and induces apoptosis [20]. Moreover, tumors treated with ONC201 exhibit an increased accumulation of activated immune cells, particularly CD3+ and activated NK+ cells [21].

Several preclinical studies have used a PC cell line-derived subcutaneous, orthotopic transplantation tumor, patient-derived subcutaneous/orthotopic xenografts (PDXs), and genetically engineered mouse models (GEMMs). However, the cell line or PDX-derived immunodeficient mouse models do not adequately reflect the actual growth environment of PC. It is believed that the GEMM model LSL-KrasG12D/+; Trp53fl/+, Pdx1-Cre (KPC) closely resembles pancreatic tumors [22]. Further, the KPC model has demonstrated clinical relevance in predicting the activity of immunotherapies [23].

To maximize the antitumor effects of L_GEM we combined it with ONC201 and evaluated their efficacy in-vitro and in a syngeneic Kras mutated xenograft PC mouse model. During in vitro studies, the combination of ONC201 with L_GEM increased the G2/M phase cell cycle arrest in MIA PaCa-2 cells. In the allograft study, the combination of L_GEM with ONC201 showed more significant results than either monotherapy, resulting in a significant reduction in tumor growth and demonstrating a favorable toxicity profile. In conclusion, the coadministration of L_GEM and ONC201 showed a synergistic effect in inhibiting PC.

## Results

### AKT and ERK pathways are active in PDAC tissues of patient samples

**Fig. 1:**
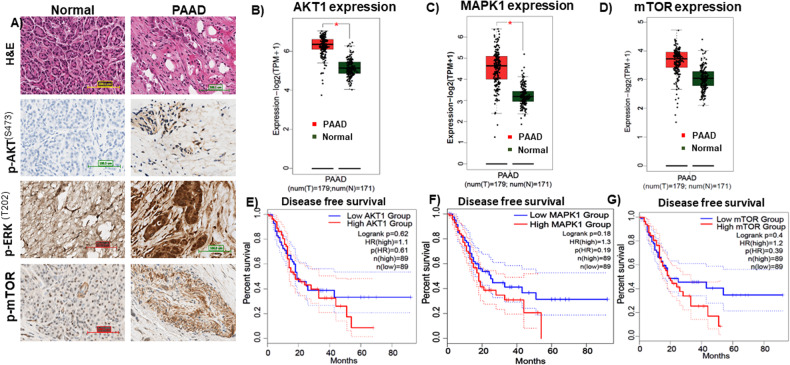
The AKT and ERK pathways are active in pancreatic adenocarcinoma (PAAD) tissue. **A** Representative images of H&E, p-AKT, p-ERK, p-mTOR in PAAD cancerous tissue or its surrounding non-tumor tissue by immunohistochemistry. The first panel shows the tissue architecture of PAAD; the second panel shows the representative images for p-AKT; the third panel shows the p-ERK; and the fourth panel shows p-mTOR expression in normal pancreatic tissues and PDAC (magnifications: ×40). **B**–**D** On the GEPIA2 web tool, AKT1, MAPK1, and mTOR expression levels were compared between PAAD and a normal population (*n* = tumor 197; normal = 171). AKT1 and mTOR genes had significantly higher expression levels in PAAD cancer specimens than in normal samples (*p < 0.01), whereas mTOR expression was not significantly different. Red color denotes tumor tissues, and green color means normal tissues. **E**–**G** The survival data of patients with PDAC demonstrate that individuals with elevated expression levels of AKT1, MAPK1, and mTOR have worse survival rates in comparison to those with lower expression levels. Solid blue color represents low expresstion and solid red color represents high expression.

PDAC patient tissues showed significantly higher levels of p-AKT ^(Ser473)^, p-ERK ^(T202/T204)^, and p-mTOR compared to surrounding non-cancerous tissues (Fig. 1A). The first panel shows the tissue architecture of PDAC, the second panel shows a representative IHC image of p-AKT ^(Ser473)^, the third panel shows a representative IHC image of p-ERK ^(T202/T204)^, and the fourth panel shows a representative IHC image of p-mTOR expression in normal pancreatic tissues and PDAC.

Using the GEPIA2 web tool, AKT1, MAPK1, and mTOR expression levels were compared between PDAC and a normal population (for tumor tissues, *n* = 197, and for normal tissues, *n* = 171) (Fig. 1B–D). AKT1 and MAPK1 genes had significantly higher expression levels in PDAC cancer samples than in normal samples (*p < 0.01), whereas mTOR expression was not significantly different. Importantly, the high expression of these genes is associated with significantly reduced survival time when compared to low-expressing groups (Fig. 1E–G). In Fig. 1, red denotes expression in tumor tissues, and green denotes expression in normal tissues.

### L-GEM and ONC201 synergistically inhibited pancreatic cancer cell proliferation in vitro

**Fig. 2:**
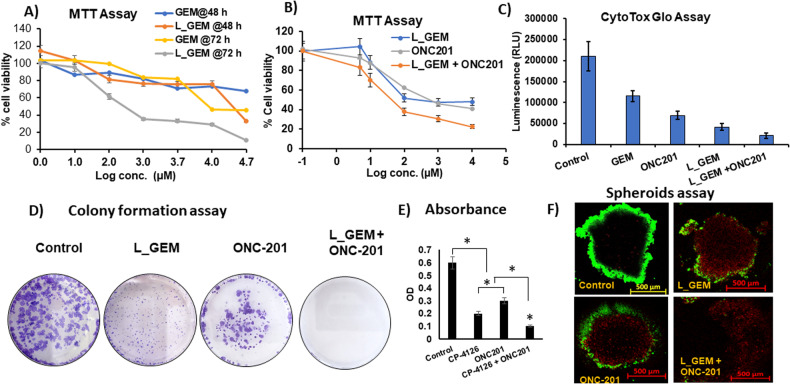
The combination of ONC201 with GEM analog (L_GEM) efficiently reduces the viability and tumorigenic potential of pancreatic cancer cells MIA PaCa-2. **A** MTT-based cytotoxicity assay of gemcitabine (GEM) and gemcitabine elaidate (CP-4126, L_GEM) at different concentrations and time points. MiA PaCa-2 cells were treated with increasing concentrations of compounds for 48 or 72 h. Percentage cell viability was measured by using MTT, in which DMSO-treated cells served as a control (100%). **B** Cells were exposed to ONC201 in combination with L_GEM for 72 h then cell viability was determined by MTT assay. (Data are expressed as mean ± SD (*n* = 5) *p < 0.05). **C** CytoTox glo assay after 48 h treatment with drugs. **D** Colony formation assay: MIA PaCa-2 cells after various treatments formed fewer colonies than control cells. **E** The columns represent the mean colony number for each group from at least three independent experiments. Data are expressed as mean ± SD. **F** Tumor spheroid assay: cells were exposed to ONC201 in combination with L_GEM for 48 h and then allowed to grow for 14 days. Calcein AM assay was used to distinguish live (green) and dead cells (red) scale bar 500 µM.

We compared the cytotoxicity effects of lipid-GEM conjugate (L_GEM) with GEM in MIA PaCa-2 cells utilizing an MTT assay. We selected these cells because prior studies have revealed that the MIA-PaCa-2 cell line includes constitutively activated AKT [24]. Each of the drugs displayed a dose-dependent cytotoxic effect (Fig. 2A). After 48 h of GEM exposure to MIA PaCa-2 cells, the half inhibitory concentration (IC_50_) was observed at 10 ± 1 µM (Fig. 2A). At the same time point, MIA PaCa-2 cells exposed to L_GEM showed an IC_50_ concentration of 1.0 ± 0.2 µM. After 72 h of treatment, a 50% reduction in cell viability compared to control cells was achieved at 1 µM and 340 nM for GEM and L_GEM, respectively. At both 48 and 72 h, the cytotoxicity of L_GEM was significantly higher than GEM.

We performed a cytotoxicity assay on MIA PaCa-2 cells using L_GEM and ONC201 combination therapy. We observed that L_GEM was more toxic than ONC201 at all tested concentrations (Fig. S1A). Further, we observed significantly enhanced cytotoxicity in the combination treatment compared to either monotherapy at 72 h (Fig. 2B, IC_50_ = 200 nM *p < 0.05). Further, combination index values between L_GEM and ONC201 were calculated using the Chao and Talalay method. CI values were <1 for the combination at all tested concentrations, indicating in vitro synergism between L_GEM and ONC201 in PC cells (Fig. S1B).

In the CytoTox Glo™ Assay, we observed that both L_GEM and ONC201 treatment induced high cytotoxicity at their IC_50_ concentrations (Fig. 2C). Intriguingly, treatment with combination led to much higher levels of death-cell protease release in MIA PaCa-2 cells. The combination of L_GEM and ONC201 inhibited colony formation, tumor spheroid growth, and invasion of MIA PaCa-2 cells more effectively than either drug alone. Figure 2D shows almost no colonies in the samples treated with the combination of L_GEM and ONC201. At the same time, ONC201 alone showed a significant decrease in colonies compared to non-treated samples. Figure 2E shows that compared to the control group (optical density (OD) 0.6 ± 0.01), the OD values of monotherapy treatment groups were 0.2 ± 0.02. As such, combination treatment inhibited the colony formation of cells better than either drug alone, which was consistent with the results from the cell viability assays.

Tumor spheroids are formed by CSCs within a tumor/cancer cell line and are correlated with cancer metastasis and aggressiveness [25]. An evident disruption of the architectural structure of the spheroid population was observed in the tumor spheroids exposed to L_GEM and ONC201 compared to control spheroids. All the treated spheroids also showed a decrease in cell viability, as indicated by decreased Calcein AM staining (green) and increased propidium iodide (PI) staining (red) (Fig. 2F). The combination exhibited the highest cytotoxicity resulting in dead cells distributed across the inner core of spheroids and the disruption of spheroid structural integrity.

### L_GEM and ONC201 combination arrests MIA PaCa-2 cells in the G2 phase and induces apoptosis more effectively than their individual drugs

**Fig. 3:**
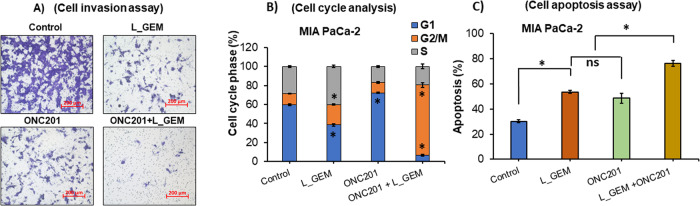
Treatment with ONC201 and L_GEM efficiently reduces invasion and induces apoptosis of MIA PaCa-2 cells. **A** Matrigel invasion assay: after treatment with L_GEM and its combination with ONC201, significantly reduced the population of the invaded cells in MIA PaCa-2 cells. **B** Data from cell cycle assay by flow cytometry using MIA PaCa-2 cells. There was a significant difference in the cell cycle arrests in L_GEM in combination with ONC201 compared with untreated cells. Data are expressed as mean ± SD. **C** Data for apoptosis assay suggest that combination treatment significantly induced apoptosis in MIA PaCa-2 cells.

As growing cancer cells try to leave the main growth site, they must stick to nearby cells and invade the surrounding tissue [26]. We used the Transwell migration and invasion assays to analyze the ability of single cells to directionally respond to various chemo-attractants in the presence of different inhibitors. L_GEM, ONC201, and their combination decreased the extent of migration of MIA PaCa-2 cells across the Transwell insert membrane compared to control cells (Fig. 3A). In line with the above results, the combination treatment more significantly decreased migration than either monotherapy.

Cell cycle analysis suggests that upon treatment of MIA PaCa-2 cells with the combination of L_GEM and ONC201, they were primarily found in the G2 phase (Figs. 3B and S2). The G2 phase arrest occurs due to DNA damage, insufficient nutrients, or signaling from growth factors. The cells will remain in the G2 phase until the issue is resolved and normal cell division can resume. If the damage is irreparable, the cells may undergo apoptosis. In the untreated control cells, the percentage of cells in the G1, G2/M, and S phases were 59.72 ± 8.6%, 11.82 ± 3%, and 28.42 ± 5.6%, respectively. We found that upon L_GEM treatment, 40.3 ± 4.6% of cells were arrested in the S Phase, while 21.37 ± 6.2% were found in and G2 phase (Fig. 3B). After treatment with ONC201, 72.31 ± 7.6% of cells were found in the G1 phase, whereas their population in G2/M and S phases was 10.93 ± 2.2% and 16.71 ± 3.2%, respectively. In the combination-treated cells, we surprisingly observed 74.12 ± 11.6% of the population in the G2 phase, while 6.53 ± 1.5% and 19.36 ± 5.6% cells were observed in the G1 and S phases, respectively.

Apoptosis is a crucial regulator of tumor growth [9]. L_GEM and ONC201 were found to induce apoptosis of cancer cells in a dose-dependent manner; therefore, we next evaluated the effects of L_GEM combined with ONC201 on apoptosis of MIA PaCa-2 cells (Fig. S3). As shown in Fig. 3C, after 24 h of drug treatment, the combination induced apoptosis more significantly than single agents. In the untreated control MIA PaCa-2 cells, the percentage of Annexin V-positive cells was 30.13 ± 2. In cells treated with L_GEM and ONC201 alone, these percentages increased to 53.5 ± 5.35 and 48.6 ± 8.3, respectively. In cells treated with the combination of L_GEM and ONC201, the percentage of apoptotic cells was 76.30 ± 8.6.

**Fig. 4:**
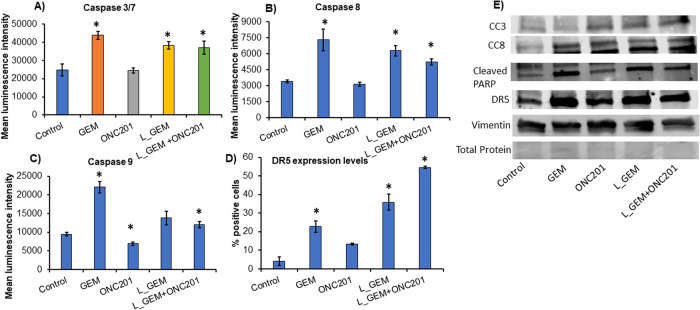
L_GEM and ONC201 induce cell apoptosis by different mechanisms. **A**–**C** Caspase activity assay. MIA PaCa-2 cells were treated with various compounds for 24 h and mean luminescence intensity reflecting Caspase 3/7, Caspase 8 and Caspase 9 activities were measured (*n* = 3, *p < 0.05). The GEM induced cell apoptosis via upregulating Caspase 3/7, caspase 8, and caspase 9 activities. While ONC201 did not show any change in Caspase activity. **D** Cells after treatment with various compounds were analyzed for DR5 expression using Flow cytometry. GEM and ONC201 both increased DR5 expression. However, the upregulation was highest in combination treatment (S.D. ± mean, *n* = 3, *p < 0.05). **E** Western blot analysis for proteins cleaved Caspases 3/8, cleaved PARP-1, DR5, and vimentin. Total protein among different groups were normalized with ponceau S staining.

In Caspase 3/7 activity assay, we observed that GEM and L_GEM induced mean luminescence intensity (ΔMLI) values of 43,826 ± 2259 and 38,298 ± 2055, respectively, indicating higher Caspase 3/7 activity at their IC_50_ (Fig. 4A, B), ONC201 did not affect this activity (ΔMLI 24,486 ± 1431). Therefore, in the group treated with L_GEM combination with ONC201 (at IC_50_ levels), the ΔMLI value 37,079 ± 3526 was not significantly different from L_GEM alone treatment (Fig. 4A). Further, Western blot analysis for cleaved Caspase 3 and 8 (CC3/8) showed increased signal when treated with ONC201 and L_GEM but not with GEM (Fig. 4E). Cleavage of PARP-1 by Caspase protein may be an important event in the apoptosis induction. Western blot for cleaved PARP-1 protein revealed that ONC201 did not increase its expression. However, GEM or L_GEM treatment showed a non-significant increase in cleaved PARP-1 expression, and its combination with ONC201 did not further increase it (Fig. 4E). These results support the hypothesis that ONC201 treatment does not induce apoptosis but lowers the threshold for the apoptotic pathways in MIA PaCa-2 cells. Similarly, Caspase 8 and 9, which are upstream of Caspase 3 and 7, were also not induced by ONC201, while GEM and L_GEM significantly enhanced their activity compared to control cells (Fig. 4B, C). These results indicate that ONC201 reduces tumor cell growth independent of apoptosis.

GEM is known to enhance TRAIL-induced cell death by upregulating DR5. The activation of the ATF4/CHOP-mediated integrated stress response (ISR) pathway by ONC201 results in DR5/TRAIL-mediated apoptosis. In vehicle-treated cells, DR5-positive cell population percentage was 4.1 ± 2.2. In GEM-treated cells, it was 22.8 ± 3.1, whereas, in L_GEM-treated cells, it was 35.9 ± 4.3 (Fig. 4D). The DR5-positive cell population increased by 13.1 ± 0.5% with ONC201 treatment alone and by 54.6 ± 0.7% with L_GEM treatment, indicating a modest impact of ONC201 on DR5 expression. Further, DR5 protein expression pattern when analyzed with Western blot also showed a similar pattern confirming these results (Fig. 4E).

### Mapping of bioenergetics in pancreatic cancer cells

**Fig. 5:**
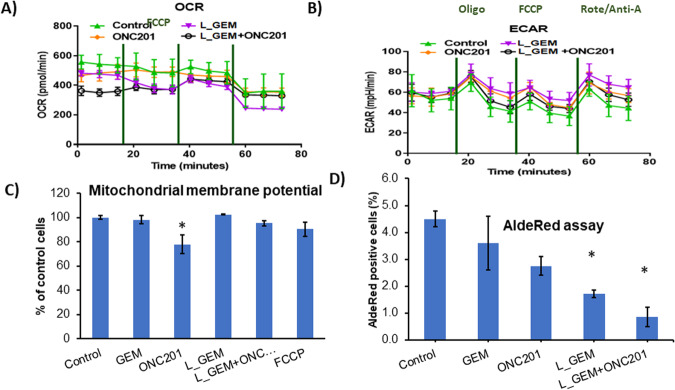
Mitochondrial stress test and glycolysis test in drug-treated MIA PaCa-2 cells. To evaluate mitochondrial function, cells were injected with oligomycin (oligo), carbonyl cyanide-4-(trifluoromethoxy)phenylhydrazone (FCCP), and rotenone/antimycin-A. **A** Quantification of the basal oxygen consumption rate (OCR), proton leak, maximal OCR, ATP-linked OCR, and spare capacity in control and treated mitochondria. **B** Extracellular acidification rate with or without treatments for 24 h. Values are expressed as mean ± SEM of three independent experiments conducted in triplicate on each treatment group. **C** Mitochondrial potential determined by flow cytometry—Tetramethylrhodamine, ethyl ester, perchlorate (TMRE) staining. **D** ALDH positive cell percentage in MIA PaCa-2 cells post 24 h treatment with various compounds (*n* = 3, *p < 0.05).

To better understand the change in metabolic and energetic requirements of MIA PaCa-2 cells after treatments, cells were analyzed in real-time using a Seahorse Extracellular Flux (XF) 96-well Analyzer. Both cellular oxygen consumption rate (OCR), resulting from oxidative phosphorylation (OXPHOS), and extracellular acidification rate (ECAR), associated with glycolytic metabolism (glycolysis), were simultaneously monitored (Fig. 5A, B). OCR and ECAR were assessed both at baseline and following injection of the drugs. The oxygen concentration changes calculated and reported as OCR for basal cells were greatest for other groups (Green, triangles) and lowest for the L_GEM and ONC201 combination group (Black, circles), as shown in Fig. 5A. An elevation in glycolytic activity is frequently reported as a compensatory mechanism, therefore we also measured the ECAR in the culture media of these cells. According to the data presented in Fig. 5B, it can be observed that the control cells exhibited the lowest ECAR, whereas the L_GEM cells demonstrated the highest ECAR values. Interestingly, the combination treatment showed no increasing effect on ECAR. These findings suggest that under the stress conditions of monotherapy, MiaPaCa-2 cells transition to the glycolytic pathway; however, the combination therapy prevents this non-mitochondrial energy production.

We determined whether the membrane potential of mitochondria is affected by treatments [27]. We observed that the fluorescence intensity of mitochondria was not affected by GEM or its analog L_GEM (Fig. 5C). However, in cells treated with ONC201, the fluorescence intensity of mitochondria was reduced to ~80% compared with the controls. Interestingly, we found that cells treated with the combination of L_GEM and ONC201 showed slightly less loss in mitochondrial membrane potential than cells treated with ONC201 alone. Many PC cell lines, including MIA PaCa-2 and BxPC3 cells, also contain an AldeRed+ population with stem cell properties [23]. We, therefore, determined the effects of drug treatments on the proportion of AldeRed+ cells in vitro (Fig. S4). Treatment with ONC201 (2.75 ± 0.6%) and L_GEM (1.70 ± 0.3%), but not with GEM (3.6 ± 1.1) significantly decreased (*p* < 0.01) the percentage of AldeRed^+^ cells (Fig. 5D). The percentage of AldeRed^+^ cells in L_GEM and ONC201 combination-treated group was further significantly decreased (*p* < 0.01) to 0.85 ± 0.1%.

### L_GEM and ONC201 combination effectively inhibits the growth of KPC tumor-bearing syngeneic mice

**Fig. 6:**
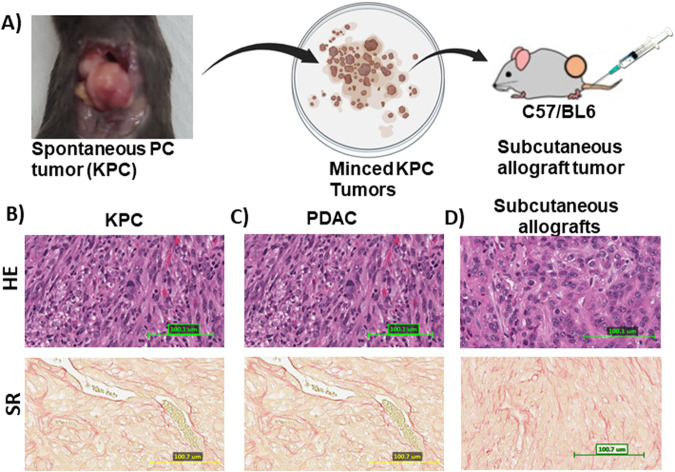
Schematic representation of in vivo drug testing using syngeneic subcutaneous KPC allografts. **A** Tamoxifen-induced spontaneous tumor-bearing KPC mouse. The harvested tumor was grafted subcutaneously in C57/BL6 mice flank and randomized for treatments. Representative image of Hematoxylin and eosin, and Sirius red staining of KPC tumors (magnification, ×20; scale bar, 100 μm). **B**–**D** Representative image of Hematoxylin and eosin (upper panel), and Sirius red (lower panel) staining of KPC tumors (magnification, ×20; scale bar, 100 μm).

**Fig. 7:**
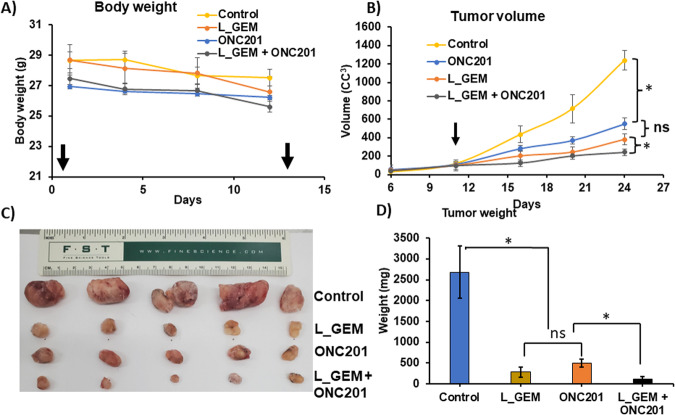
L_GEM and ONC201 combination therapy more effectively inhibits tumor growth than their monotherapies after systemic administration in KPC pancreatic cancer allograft-bearing mice. **A** Effect of treatments on body weight change. The weight of each mouse was measured once a week. **B** Effect of therapy on tumor volume (*n* = 5). The volume of each tumor was measured every week. The average tumor volume in control, L_GEM (20 mg/kg), ONC201 (20 mg/kg), and the combination-treated group was plotted (mean ± SD, *n* = 5, *p < 0.05). **C** Tumors were harvested from different treatment groups after completion of the therapy. **D** Tumor weight (mean ± SD, *n* = 5, *p < 0.05).

We used PC tumor tissues from tamoxifen-induced KPC mice to create syngeneic subcutaneous tumors (Fig. 6A). KPC mouse tumors recapitulate many of the salient clinical and histopathological features of human disease (Fig. 6B–D). Animals bearing tumors were treated with doses of L_GEM and ONC201 at 20 mg/kg and 15 mg/kg, respectively. The selected doses showed no severe toxicity, as evident by the appearance, physical activity, and body weights of treated animals (Fig. 7A).

**Fig. 8:**
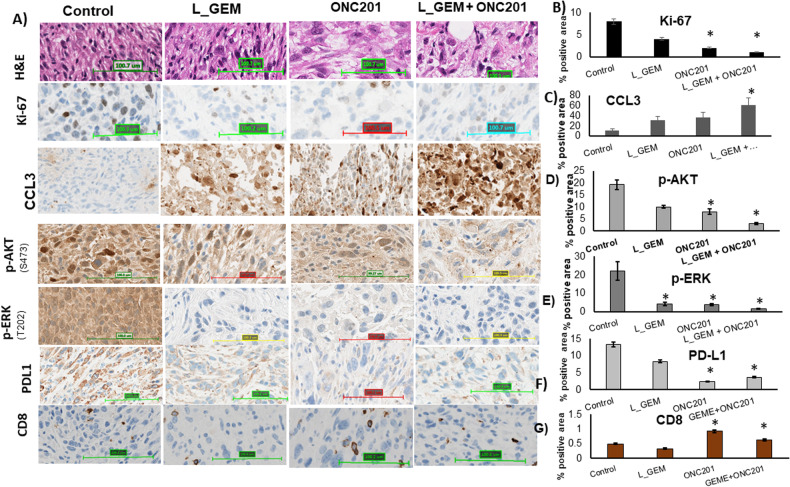
Immunohistochemistry (IHC) staining of mice tumor samples. After systemic delivery in KPC pancreatic cancer allograft-carrying mice, treatment with the combination of L_GEM and ONC201 reduced tumorigenic genes more effectively than each individual treatment. **A** Representative HE, Ki-67, CCL3, p-AKT, p-ERK PDL1, and CD8 staining from tumor tissues from treatment groups. All images were obtained at 40×. Quantification of positive staining of (**B**) Ki-67, (**C**) CCL3, (**D**) p-AKT, and (**E**) p-ERK, (**F**) PDL1, and (**G**) CD8 from the samples (*n* = 3, *p < 0.05 compared to control).

As shown in Fig. 7B, a significant increase in the tumor volume occurred on the 11th day after transplantation. Consistent with cell viability, cell proliferation, and apoptosis experiments, the in vivo efficacy study showed that L_GEM and ONC201 monotherapies had no significant effect on tumor growth inhibition (Fig. 7B). Compared to L_GEM monotherapy, the combination of ONC201 and L_GEM significantly reduced the tumor size (Fig. 7C, D). Representative tumor images of isolated tumors are shown in Fig. 7C. The number of Ki67-positive cells in tumor tissues of both control and treatment groups showed that the combination significantly suppressed tumor cell proliferation (Fig. 8A, B). In addition, ONC201 inhibited the expression of p-ERK^(T202/T204)^ and p-AKT ^(Ser473)^ in tumors and upregulated the expression of cleaved Caspase 3, thereby inhibiting the growth of these breast cancer cells in vivo (Fig. 8A, C–E).

The PD-1 receptor (programmed death 1) is primarily expressed on T cells, and its physiologic interaction with PD-L1 on cancer cells result in T cell function suppression [28]. In pancreatic cancer cells, KRAS is known to promote the expression of PD-L1 through reactive oxygen species (ROS)-mediated growth factor signaling [29]. So, we were interested in assessing how ONC201 might affect PD-L1 expression and subsequent CD8 cell tumor invasion. We observed a significant decrease in PD-L1 expression following treatment with ONC201 but not with L_GEM (Fig. 8).

We next tested whether L_GEM and ONC201 might have different impacts on T cell functions, such as their ability to infiltrate the tumor tissues. As shown in Fig. 8A, G, the administration of ONC201 alone significantly increased the number of tumors infiltrating CD8+ T lymphocytes compared to the control samples. L_GEM treatment significantly reduced the number of CD8+ T cells in the tumor tissues, whereas their combination did not show such a suppressive effect.

## Discussion

The majority of individuals (~80% to 85%) diagnosed with pancreatic cancer are deemed ineligible for surgical intervention due to the presence of advanced or metastatic disease. Chemotherapy is, therefore, the only treatment available to most people with PC. However, the development of chemoresistance often leads to poor therapeutic outcomes. Several combination therapies have been tested to overcome GEM resistance, but most have shown mixed results. Among these, only two combination treatments, one is 5-fluorouracil (5-FU)/leucovorin with irinotecan, oxaliplatin (FOLFIRINOX), and the other is GEM with nab-paclitaxel have proven to be the best for metastatic PC [30]. However, the effectiveness of these chemotherapies is severely constrained by high-grade toxicity and the emergence of chemoresistance.

The underlying mechanisms for the development of GEM resistance revolve around the upregulation of transcription factors, enzymes, and signaling pathways involved in nucleoside metabolism [13]. Cumulative evidence from past studies suggests that enhancing GEM metabolic stability and reducing its non-specific distribution is a viable strategy for increasing effectiveness and reducing side effects [31]. GEM’s rapid degradation could be prevented by bioconjugation and nanomedicine techniques. We have previously shown that GEM’s efficacy could be improved by decreasing its metabolism, controlling its accumulation at the tumor site, and rationally combining it with chemosensitizing drugs [13, 30, 32, 33]. GEM loading into nanoparticles is difficult, and bioconjugation with large molecules like polymers is a complex process that may face issues with the scaling up. Another bottleneck for GEM’s efficacy is its uptake by tumor cells. GEM is a hydrophilic nucleoside, and its cellular uptake depends on membrane nucleoside transporter hENT1 expression. L_GEM (gemcitabine-5’-elaidic acid ester) prodrug used in this study is a GEM analog that is protected from rapid degradation and can enter cells in a transporter-independent manner due to its conjugation with a fatty acid derivative [34].

To explain the decreased cell survival observed during co-treatment with ONC201 and L_GEM, we reasoned that ONC201 might lower the apoptotic threshold. We investigated this by measuring the activities of Caspases 3, 7, 8, and 9 in MIA PaCa-2 cells after 48 h of treatment. The intrinsic apoptotic pathway is initiated by Caspase 9 by inducing mitochondrial stress. On the other hand, Caspase 8 mediates the extrinsic apoptotic pathways that are mainly activated by cell surface death receptors. These two initiator Caspases (8 and 9) activate the executioner Caspases 3 and 7, which deteriorate cell structures to induce apoptosis [35]. Compared to vehicle treatment, ONC201 treatment did not affect Caspase 3/7 activities in cells. However, GEM or L_GEM treatment caused a significant increase in Caspase 3/7 activity (Fig. 4A). Further, GEM/L_GEM significantly enhanced Caspase 8 (external apoptosis) and 9 (internal apoptosis) activities in MIA PaCa-2 cells but ONC201 did not show any induction of Caspase activity (Fig. 4B, C). Similar observations were reported earlier when doxycycline did not affect the Caspase activity but primed cancer cells for apoptosis by GEM [36]. It also implies that ONC201 executes cell death by some mechanisms other than Caspase activation, which has been reported by Greer et al. in breast cancer cells [37]. The TNF family member TNF-related apoptosis-inducing ligand (TRAIL) is a potent inducer of apoptosis. TRAIL binds to DR5 and activates the Caspase-8-initiated apoptosis [38]. GEM has been linked to a synergistic cytotoxic effect with TRAIL, and it was found that pretreatment with GEM enhanced TRAIL-induced apoptosis accompanied by DR-5 up-regulation [39].

The overexpression of several IAP family proteins, including cIAP1 and XIAP, in pancreatic cancer cells makes them resistant to TRAIL-induced extrinsic apoptosis. cIAP-1 and XIAP inhibit TRAIL-induced apoptosis by preventing the cleavage of Caspase 3, 7, or 9, thereby preventing subsequent apoptotic events. These cells can, however, still undergo type II extrinsic apoptosis, which amplifies cell death signaling via the mitochondria. Further, our results indicate that GEM or L_GEM treatment increased the percentage of DR5-positive cell population in MIA PaCa-2 cells (Fig. 4D). These findings also imply that the combination can be used successfully as chemotherapy to overcome TRAIL-resistant defects in MIA PaCa-2 cells by increasing DR-5 expression.

Further, several signaling pathways are involved in GEM resistance, including PI3K/AKT, MAPK signaling, and IL-6/IL-6R/STAT3 pathways [40]. More than 90% of pancreatic cancers have mutant *K-Ras* that activates various downstream effector-signaling pathways, including the PI3K/AKT. Further, the PI3K pathway responds to stimuli from multiple growth factor receptors on the cancer cell surface [41]. A downstream element of this pathway is the mammalian target of rapamycin (mTOR). The PI3K/AKT/mTOR pathway is essential for many cellular functions, including growth, survival, and proliferation. Also, PI3K signaling in stromal cells modifies the tumor microenvironment (TME) to dictate disease outcomes. The high incidence of mutations in the PI3K signaling cascade, accompanied by activation of parallel signaling pathways, makes PI3K a promising candidate for drug therapy. Cui et al. demonstrated that treatment with the mTOR inhibitor everolimus inhibits the growth and activity of pancreatic cancer resistant to GEM [42]. Similarly, Gu et al. showed that miR-3178 inhibitor reduced GEM resistance in PC cells by inhibiting PI3K/AKT pathway [43]. In addition, Zheng et al. found that ERK1/2 activity also protects PC cells from apoptosis caused by chemotherapy. Using an ERK1/2 inhibitor (U0126) in combination with GEM, they showed synergistic therapeutic effects at lower doses of GEM [44].

The RAS/RAF/MEK/ERK and PI3K/AKT/mTOR signaling pathways interact and act as a compensatory mechanism when one pathway is inhibited. This supports the rationale for inhibiting both pathways simultaneously [45, 46]. Awasthi et al. recently demonstrated that combining the PI3K inhibitor MK2206 and the MAPK inhibitor trametinib with chemotherapy enhanced the antiproliferative effect in a PDX-derived preclinical PDAC tumor model [47]. Our study demonstrates that simultaneous inhibition of the PI3K and ERK pathways with ONC201 induces higher toxicity to L_GEM in MIA PaCa-2 cells.

Immunotherapies that induce T-cell responses have demonstrated efficacy against a subset of solid tumors in patients and rodents, but these treatments are ineffective against PC [48]. There may be several causes, including desmoplasia, an immunosuppressive TME, and excessive PDL-1 expression. KRAS hyperactivity has recently been demonstrated to upregulate PDL-1 expression in cancer cells, rendering these malignancies resistant to T cell therapy [49]. On the other hand, T-cell migration was significantly reduced in vitro and in vivo by GEM, explaining the poor clinical result of combined PD-1 antibody therapy in PC. Our findings are consistent with prior studies, and ONC201, but not L_GEM therapy, decreased PDL1 expression due to inhibition of KRAS signaling. Furthermore, whereas L_GEM alone prevented T cell migration into tumors when combined with ONC201, T cell existence was restored (Fig. 8).

In summary, we have shown that simultaneous inhibition of ERK/PI3K improves the effectiveness of the current chemotherapy in PC. We combined L_GEM with ONC201 and evaluated it in vitro and the syngeneic KRAS mutated xenograft PC mouse model. We used KPC mouse-derived subcutaneous models of pancreatic cancer to evaluate. In the allograft study, the combination of L_GEM with ONC201 demonstrated a favorable toxicity profile and significantly reduced tumor growth compared to either monotherapy. Furthermore, the coadministration of L_GEM with ONC201 clearly induced T-cell infiltration and inhibited PC tumor development.

## Material and methods

The details of “Materials and methods” used are provided in the Supplementary Methods section.

### Supplementary information

Supplementary Methods

Supplemental Figures.

Original Data

## Data Availability

The results shown in this manuscript were partially based on data from Gene Expression Profiling Interactive Analysis 2. The experimental data supporting this paper’s hypothesis are available from the corresponding author upon reasonable request.
