# Supplementary material for: Gemcitabine elaidate and ONC201 combination therapy for inhibiting pancreatic cancer in a KRAS mutated syngeneic mouse model
Source: Cell Death Discov. 2024 Mar 29;10:158. doi: 10.1038/s41420-024-01920-9 (PMC10980688; doi:10.1038/s41420-024-01920-9)
Supplement: Supplementary file 1 — Supplementary Methods [file 41420_2024_1920_MOESM1_ESM.pdf]

Supplementary Materials and Methods

Gemcitabine elaidate and ONC201 combination therapy inhibits pancreatic cancer in a KRAS mutated syngeneic mouse model

Virender Kumar[1], Bharti Sethi[1], Dalton W. Staller[2], Prakash Shrestha[1], and Ram I. Mahato[1,*]

[1]Department of Pharmaceutical Sciences University of Nebraska Medical Center, Omaha, NE 68198

[2]Department of Cellular & Integrative Physiology, University of Nebraska Medical Center, Omaha, NE, USA

Materials

L_GEM and ONC-201 were purchased from Medchem Express Inc. (New Jersey, NJ). KPC mice were purchased from Jackson Laboratory (Bar Harbor, ME**)**. Tamoxifen was purchased from Astatech Inc. (Bristol, PA). PDAC tissues were obtained from the Tissue bank at the University of Nebraska Medical Center (UNMC) after obtaining IRB approval.

PDAC tissue immunohistochemistry (IHC)

Phospho-Akt, phospho mTOR, and phospho-ERK protein expressions were evaluated in patients PDAC-tissue (n = 10). Tissue slides were stained with phospho-AKT (Ser473), Cat# 9271S, dilution 1:50) Cell Signaling, and phospho-ERK (Thr202/Tyr204, Cell signaling, Cat# 4370, dilution 1:200) phospho-mTOR (59Ser 2448 Santa Cruz Biotechnology. Cat# sc-293133, dilution 1:500), antibodies. The sections were scanned with an Aperio CS2 Scanning System at 40 × magnification (Leica Biosystems, Wetzlar, Germany).

Bioinformatics analysis

To compare the expression of AKT1, MAPK1, and mTOR genes in pancreatic ductal adenocarcinoma (PDAC) tissues to normal pancreatic tissue and correlate it with patient disease-free survival, we used Gene Expression Profiling Interactive Analysis 2 (GEPIA2, http://gepia.cancer-pku.cn/) (1). After a request for analysis, GEPIA2 can provide the results in the form of visual images of box plots and survival analysis.

Assessment of cell viability by MTT and CytoTox-Glo™ cytotoxicity assay

For the cell viability assay was performed MIA PaCa-2 cells by using MTT reagent. For CytoTox-Glo™ cytotoxicity assay Spent media (25 μL) was added per well to 96 well-white tissue culture-treated plates (Corning 734–1665). This was then combined with 25 μL of CellTiter-Glo reagent and mixed on a plate shaker for 3 minutes to ensure cell lysis. Luminescence was then read using a BioTek Synergy HT plate reader.

Caspase 3/7 activity assays

For Caspase-Glo® 3/7 luciferase-based assays, the spent media was removed from the wells to be analyzed on 96 well white tissue culture-treated plates (Corning 734–1665), replenishing with 100 μL of media. This was then incubated with 100 μL of Caspase-Glo® reagent for 30 minutes under gentle agitation at room temperature and protected from light. Then, luminescence was read using a molecular devices eM5 plate reader over 0-3 h. By subtracting the background, the mean luminescence intensity (ΔMLI) was calculated and plotted (n=5).

Western blot analysis

Western blot analysis was performed according to our established protocol [30].

Flow cytometry

After treatment period, cells were detached and isolation by centrifugation, cells were resuspended in 400 μl of ice-cold 5% fetal calf serum (FCS) in PBS and incubated for 30 minutes on ice with 5 μl (0.25 μg) DR5-PE-stained antibody (ebiosciences 12–9908), with 5 μl (0.5 μg) of PE stained IgG1k isotype control antibody (eBiosciences, 12–

4714), or were left untreated. Post incubation, the cells were centrifuged, washed, and fixed for 20 min in fixation buffer (eBioscience, 00-8222-49). Next, the cells were centrifuged and resuspended in 1 ml of ice-cold 5% FCS PBS, and this was repeated twice before samples were read by the BD FACS Calibur II flow cytometer.

Detection of apoptosis with flowcytometry

Cells seeded at 0.4 × 10^6 cells per well in a six-well plate were treated with drugs or equivalent control for 24 h. After removing the media, cells were harvested with a scraper, and stained for 30 min on ice with 3 µL Annexin V FITC antibody (BD Pharmingen) and washed with staining buffer twice. Next, cells were incubated for 15 min at room temperature with 2 µL propidium iodide. The samples were then analyzed using a BD FACS Calibur II flow cytometer.

Extracellular flux assay

To determine the mitochondrial and glycolytic function of pancreatic cancer cells, we used the bioenergetic function assay using Seahorse XF96Fe Extracellular Flux Analyzer from Seahorse Bioscience (North Billerica, MA). After seeding overnight, the cells (20000/well) were washed with unbuffered assay media (supplemented with 5.5 mM glucose and 4 mM L-glutamine, but no pyruvate or sodium bicarbonate) and conditioned for 1 h before measurements. After cell conditioning in the test medium, eight baseline oxygen consumption rate (OCR) and extracellular acidification rate (ECAR) measurements were taken within an hour, and the last six (most stable) readings were averaged. Using the media's estimated buffer capacity and the chamber volume in the XF96 Analyzer, the ECAR expressed in mph/ min was automatically translated to the

proton production rate (PPR) stated in pmol H^+ min^{-1}. Different levels of mitochondrial respiration and/or acid extrusion (i.e., the status of basal glycolysis) are a sign of a cell's sensitivity to inhibitors.

Measurement of mitochondrial membrane potential

The mitochondrial membrane potential of treated cells was measured using staining with tetramethylrhodamine (TMRE) and using flow cytometry. MIA PaCa-2 cells were seeded at 500,000 cells per well in a 6-well plate and treated with inhibitors at their IC_{50} for 24 h. Cells were stained with 200 nM TMRE for 15 minutes at 37 ˚C. To measure mitochondrial membrane potential, the cells were gently detached using a cell scraper and washed twice in PBS. The samples were then analyzed using a BD FACS Calibur II flow cytometer.

AldeRed assay

AldeRed assay was performed following a modified version of manufacturer's protocol. In brief, after exposure to different compounds, 100,000 cells were resuspended in a 1 ml AldeRed assay buffer. After adding 5 µL AldeRed™ reagent and briefly mixing, 300 µL of the cell suspension was immediately transferred to another tube supplemented with 5 µL N, N-diethylaminobenzaldehyde (DEAB) and mixed by pipetting. Both tubes were then placed at 37 °C for 45 min to allow the reaction to occur. Next, the cells were washed twice with AldeRed assay buffer and analyzed by flow cytometry.

Cell line xenografts

All animal studies were performed with approval with IACUC at the UNMC. Under brief anesthesia, male or female C57Bl/6J mice were injected with freshly harvested KPC tumor pieces (2-3 mm) subcutaneously into the right hind flank. Volumes of outgrowing tumors were evaluated using the formula: volume = $width^2 \times length \times ½$. When tumors reached a volume of approximately 100 mm^3, about 9–12 days post-tumor inoculation, animals were injected intravenously (i.v.) with equivalent volumes of solvent alone and served as vehicle-treated controls (vehicle, n=5). Additional groups were injected with L_GEM (20 mg/kg), ONC201 (15 mg/kg), or their combination three times a week for two weeks. During the treatment, mice were monitored for body weight and tumor volume. Tumor volumes were measured on alternate days and plotted over time. Mice were sacrificed after the treatment regimen or when they became moribund before the tumor volume reached 2000 mm^3. At the end of each experiment, blood samples, tumors, and other major organs were removed from the animals of all groups for further analysis.

Allograft tumor histology and immunohistochemistry

Tumor cell proliferation and apoptosis in tissue samples were stained with Ki-67, CCL3, p-AKT, p-ERK, PDL1, and CD8. Slides were scanned with Apiro Versa 200 (Leika, Wetzlar, Germany). The staining was quantified with Image J software.

Statistical analysis

Statistical analyses were conducted using GraphPad Prism 5 software. Differences were analyzed with a one-way analysis of variance (ANOVA) followed by Tukey's post hoc test for multiple comparisons and Student's *t*-test for two comparisons.

Data were presented as the means ± standard error of the mean. Significance was set at $P < 0.05$. *, **, and *** indicates $p < 0.05$, $p < 0.01$, and $p < 0.001$, respectively.

References

1. Feng H, Gu ZY, Li Q, Liu QH, Yang XY, Zhang JJ. Identification of significant genes with poor prognosis in ovarian cancer via bioinformatical analysis. J Ovarian Res. 2019;12(1):35.
