# Supplementary material for: Gemcitabine elaidate and ONC201 combination therapy for inhibiting pancreatic cancer in a KRAS mutated syngeneic mouse model
Source: Cell Death Discov. 2024 Mar 29;10:158. doi: 10.1038/s41420-024-01920-9 (PMC10980688; doi:10.1038/s41420-024-01920-9)
Supplement: Supplementary file 2 — Supplemental Figures. [file 41420_2024_1920_MOESM2_ESM.pdf]

Supplementary Figures

Pancreatic cancer regression by Gemcitabine elaidate and ONC201 combination therapy in a syngeneic mouse model

Virender Kumar,[1] Bharti Sethi,[1] Dalton Staller,[2] Prakash Shrestha[1] and Ram I Mahato[1,*]

[1]Department of Pharmaceutical Sciences University of Nebraska Medical Center, Omaha, NE 68198

[2]Department of Cellular & Integrative Physiology, University of Nebraska Medical Center, Omaha, NE 68198

This file includes:

Figures: S1 to S5

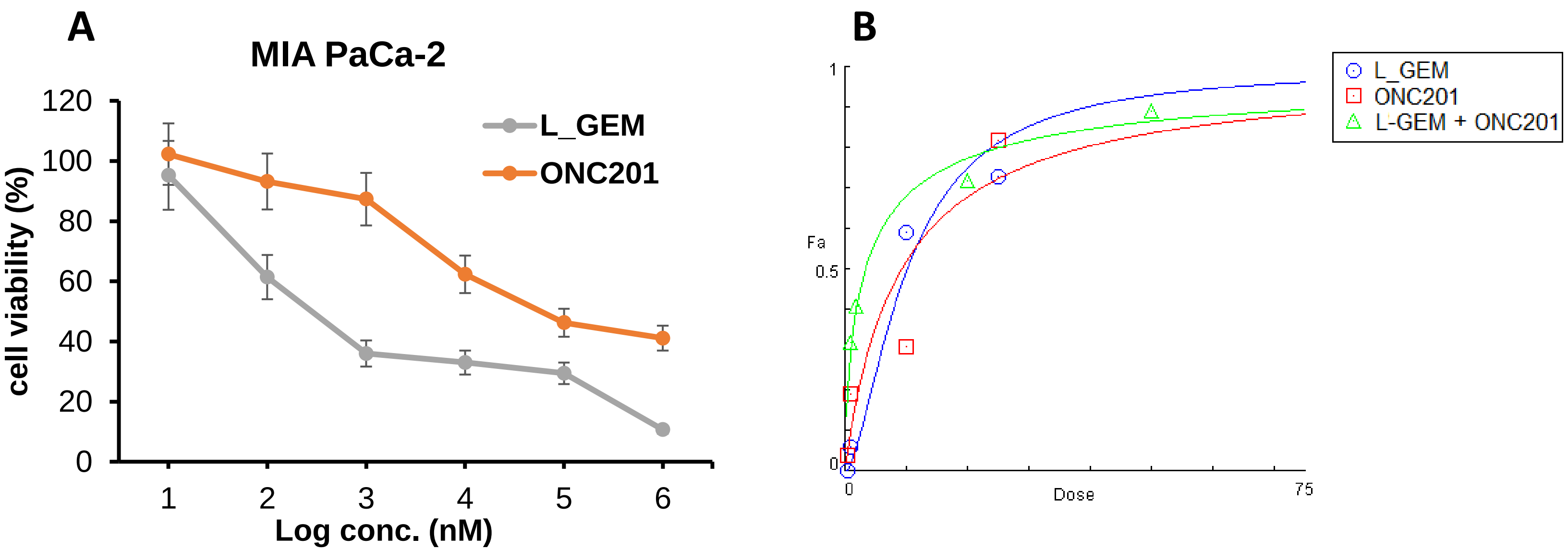

Figure S1. MTT assay in MIA PaCa-2 cells after 72 h of treatment with L_GEM and ONC201 at different concentrations. A) Cell survival after treatment. B) Combination index curve.

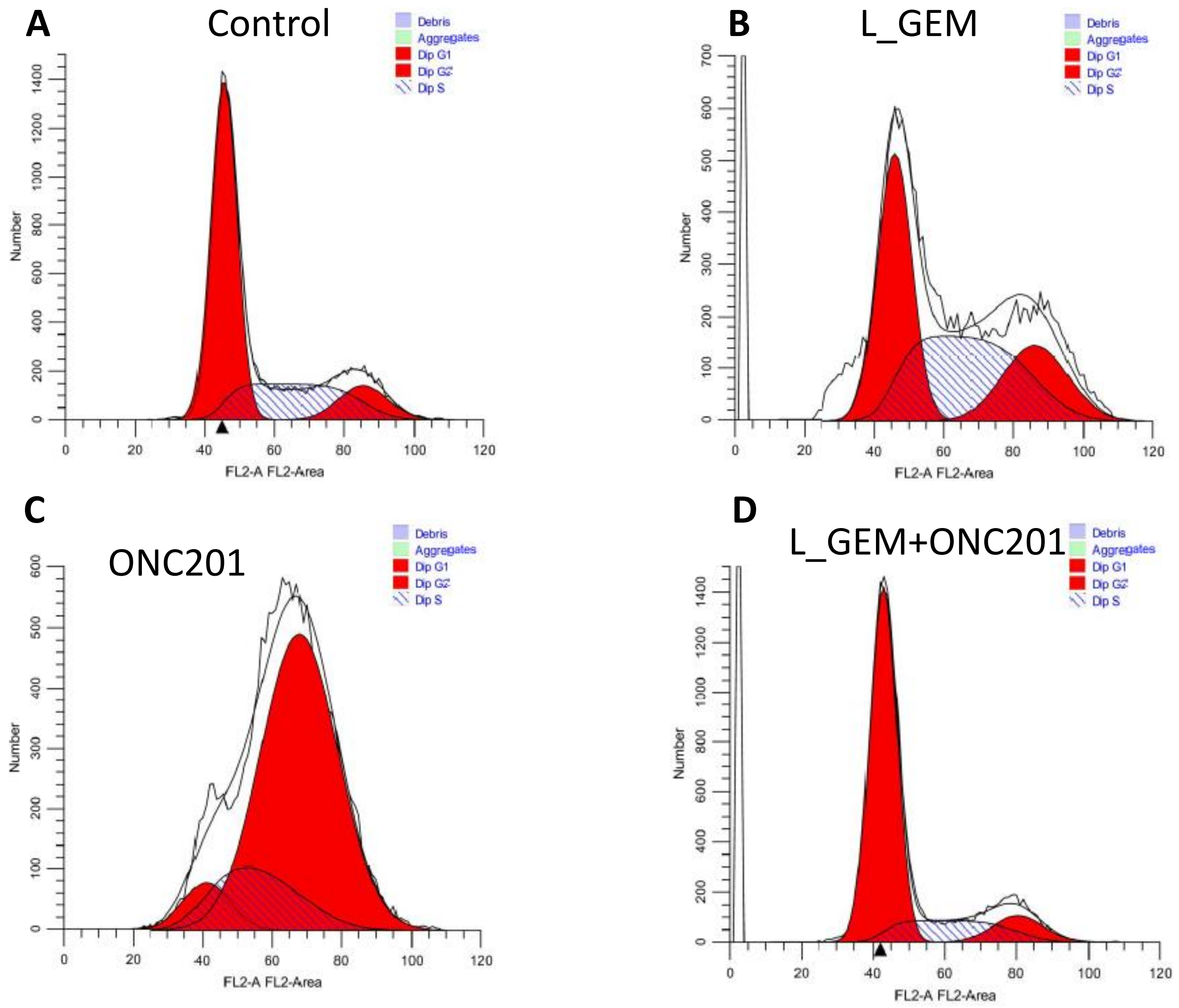

Figure S2. Representative images of MIA PaCa-2 cells in different phages of cell cycle post 24 h treatment with L_GEM and ONC201 at different concentrations, while non-treated cells were used as the control.

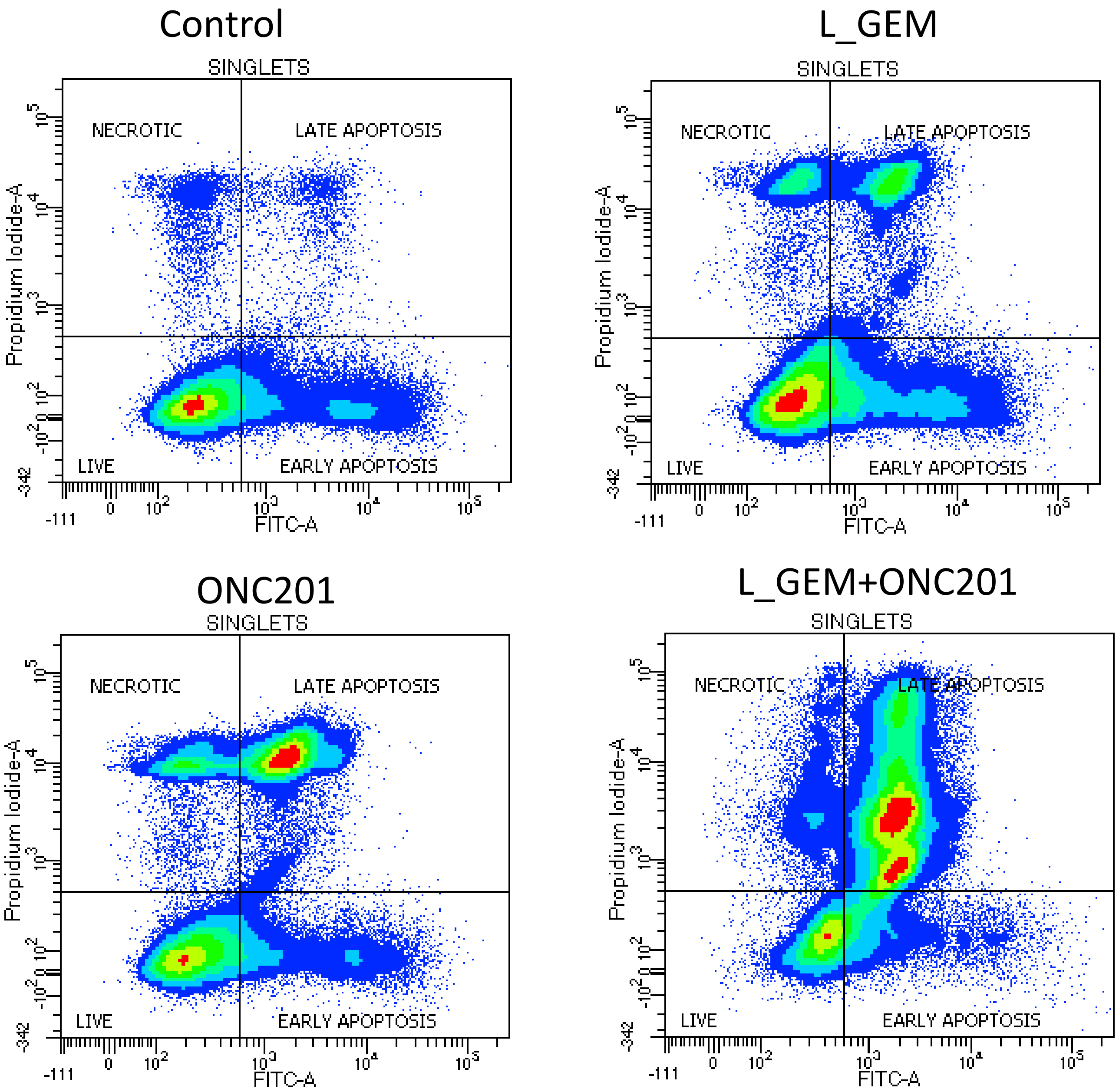

Figure S3. Representative pictures showing the percentage of MIA PaCa-2 cells that underwent apoptosis after being exposed to L_GEM and ONC201 at various doses for 24 hours, with untreated cells serving as the control.

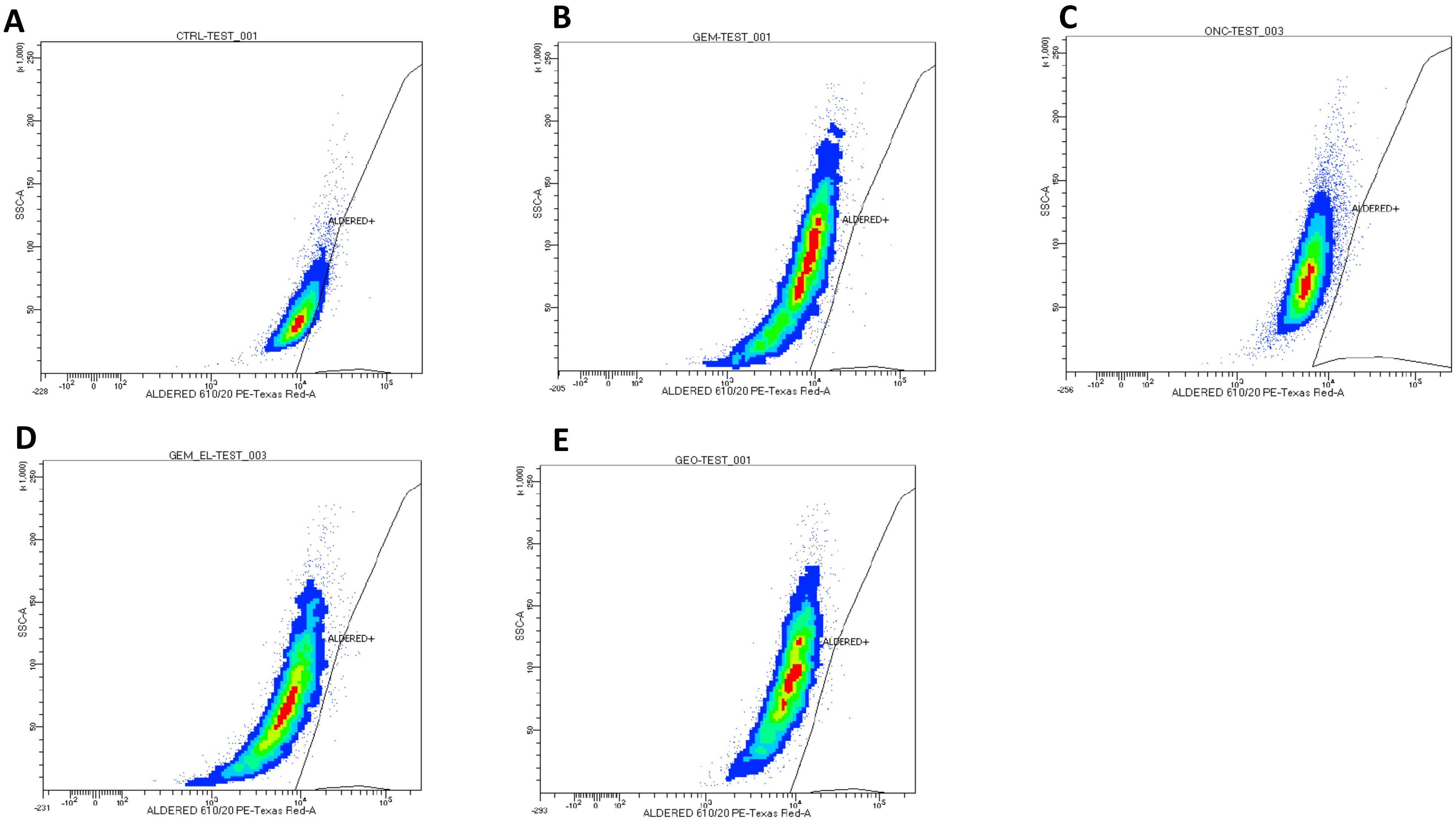

Figure S4. Images showing the proportion of ALDH-positive cells in MIA PaCa-2 cells after treatment with L_GEM and ONC201 for 24 hours at varying doses, with untreated cells serving as a control.

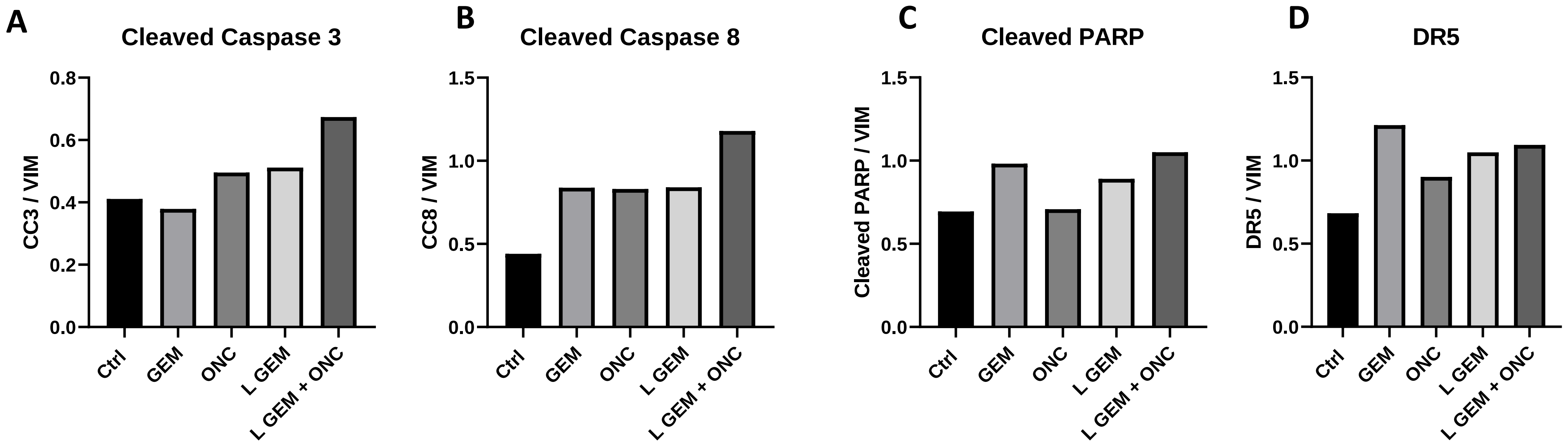

Figure S5. Quantitation of density of signals from Western blot analysis for proteins cleaved Caspases 3/8, cleaved PARP1, and DR5.
