# Supplementary figures and images for: Gemcitabine elaidate and ONC201 combination therapy for inhibiting pancreatic cancer in a KRAS mutated syngeneic mouse model
Source: Cell Death Discov. 2024 Mar 29;10:158. doi: 10.1038/s41420-024-01920-9 (PMC10980688; doi:10.1038/s41420-024-01920-9)

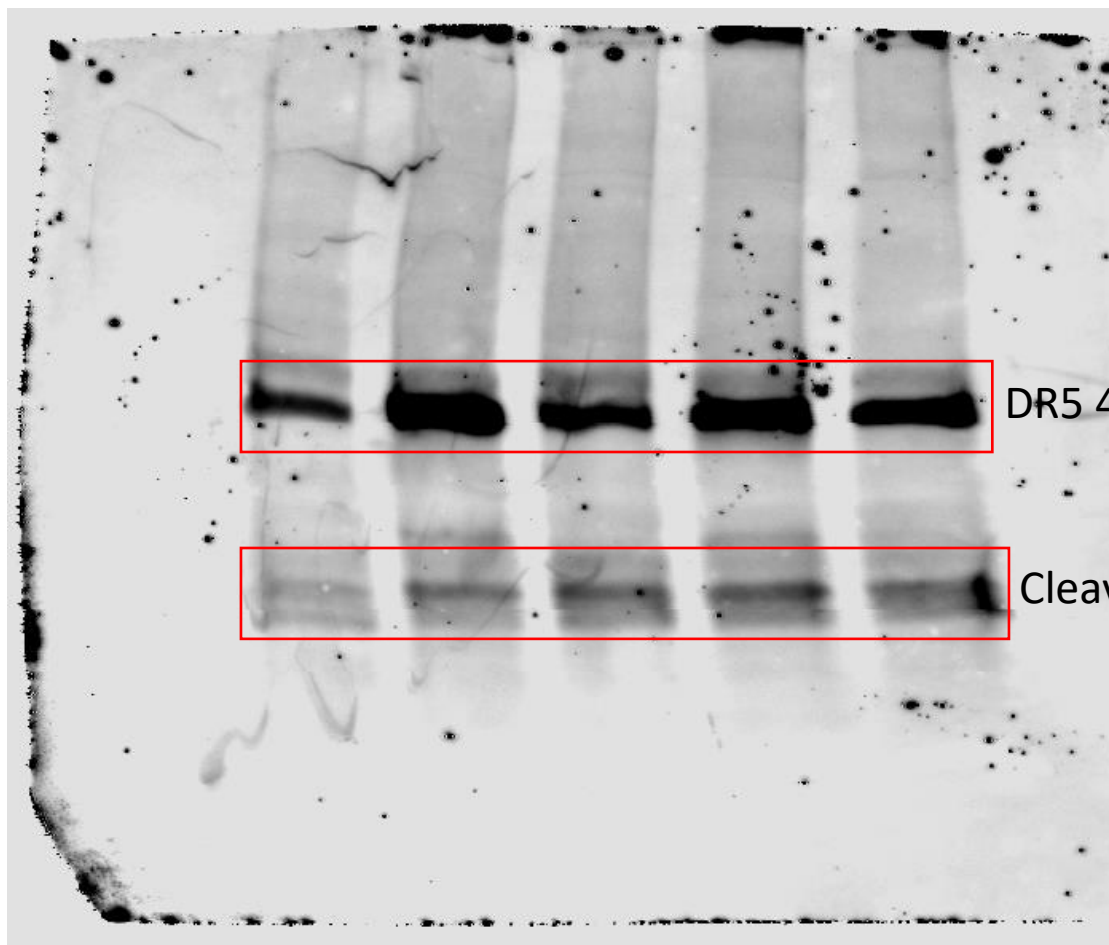

Total Protein

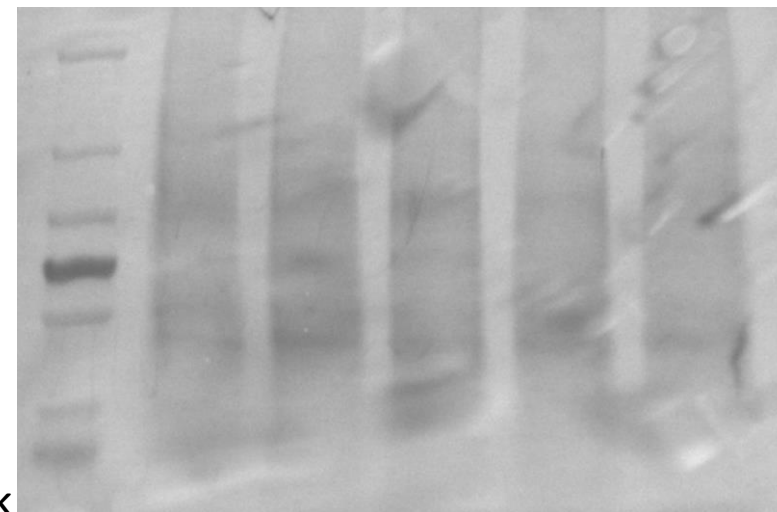

Total Protein

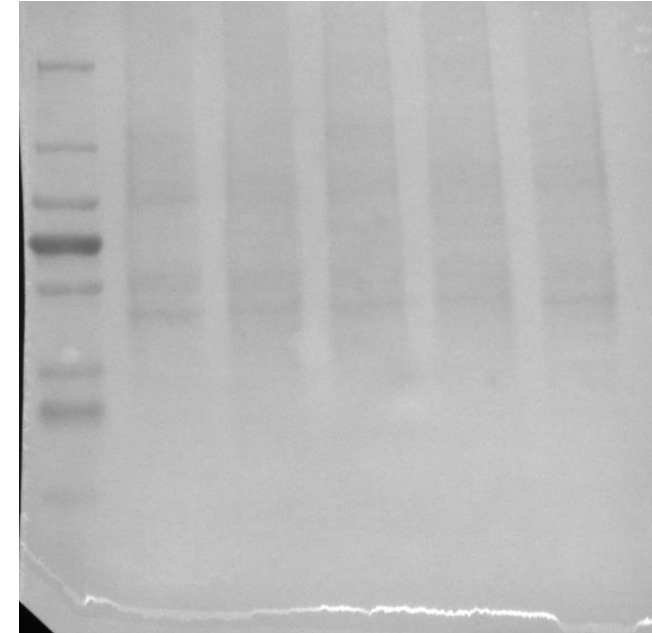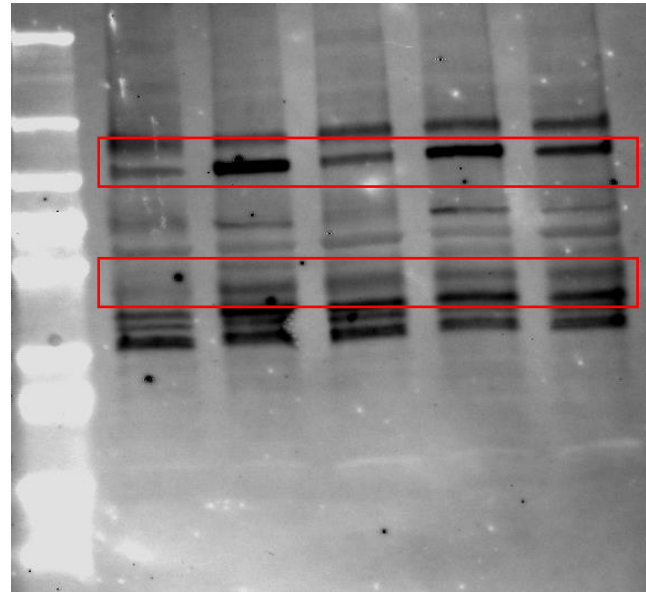

Cleaved PARP 89k

Cleaved Caspase-8 43k

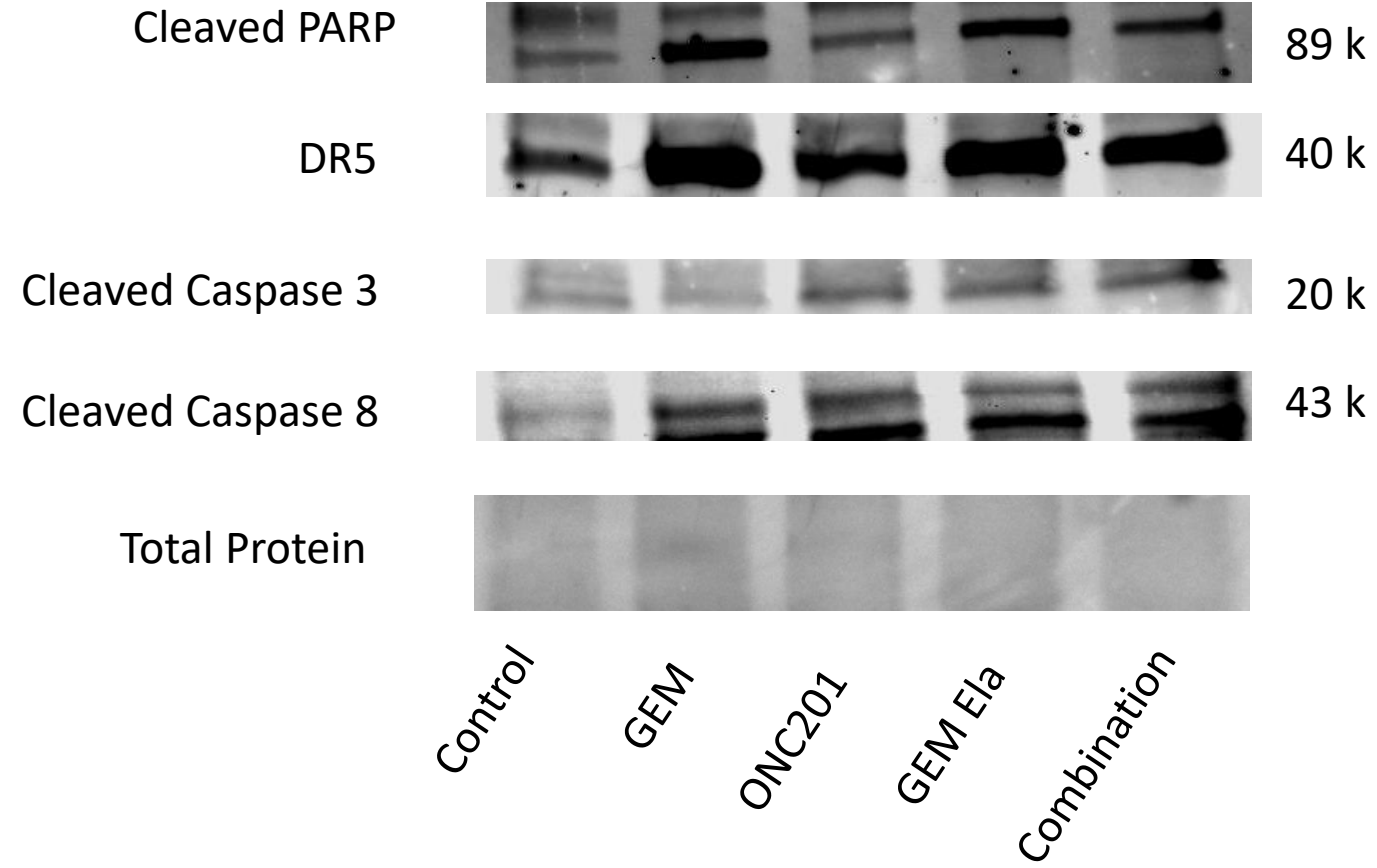

Supplement: Supplementary file 3 — Original Data [file 41420_2024_1920_MOESM3_ESM.pdf]
